# Spontaneous ovarian hyperstimulation syndrome in early singleton pregnancy with hepatic involvement: two case reports of atypical presentations

**DOI:** 10.3389/frph.2026.1872608

**Published:** 2026-06-24

**Authors:** M. J. Nuñez Burbano, Sergio Andrés Escobar, Dayanna Pinto-Martínez, Víctor S. Rangel, Ricardo García Yepes, Yaisa Maritza Castaño Toro, Jorge L. Campo

**Affiliations:** 1 Instituto de la Mujer, Hospital Universitario Mayor-Méderi, Bogotá, Colombia; 2 Universidad del Rosario, Bogotá, Colombia; 3 Clinical Investigation Group, Universidad del Rosario, Bogotá, Colombia

**Keywords:** ascites, cholelithiasis, liver diseases, ovarian hyperstimulation syndrome, pregnancy

## Abstract

**Background:**

Ovarian hyperstimulation syndrome (OHSS) is typically an iatrogenic complication of assisted reproductive techniques. Spontaneous OHSS is rare, particularly in singleton pregnancies and in the absence of known risk factors, and may present with atypical systemic manifestations such as hepatic involvement.

**Case report:**

We report two cases of spontaneous OHSS in early singleton pregnancies with hepatic involvement. The first case was a 21-year-old woman presenting with abdominal distension, ascites, and hyperbilirubinemia, with imaging revealing markedly enlarged ovaries and no evidence of primary hepatic pathology, although biliary tract findings consistent with cholelithiasis were identified. The second case involved a 23-year-old woman with abdominal pain, bilious vomiting, and elevated liver enzymes, with ultrasound findings of bilateral ovarian enlargement and cholelithiasis. In both cases, the diagnosis was established based on clinical and imaging findings after exclusion of other causes. Management was conservative, including close clinical and laboratory follow-up; however, one patient required laparoscopic cholecystectomy due to symptomatic biliary disease.

**Conclusion:**

These cases highlight that spontaneous OHSS should be considered in pregnant patients presenting with abdominal symptoms, ovarian enlargement, and abnormal liver function tests, even in the absence of assisted reproduction. The relationship between spontaneous OHSS and hepatic abnormalities remains incompletely understood, particularly in the presence of concomitant biliary disease. Further studies are needed to better characterize the hepatic manifestations, epidemiology, and underlying genetic mechanisms of this condition.

## Introduction

Ovarian hyperstimulation syndrome (OHSS) is a condition classically described as an iatrogenic complication of assisted reproductive techniques, with an estimated incidence of moderate to severe disease ranging from 1% to 5% of patients undergoing treatment (1). In rare cases, it may occur in spontaneous ovulatory cycles, particularly in the context of multiple pregnancies, hypothyroidism, or gestational trophoblastic disease. Additionally, its occurrence has been reported in patients with a family history of OHSS, in association with mutations in the follicle-stimulating hormone receptor (FSHR) gene (2–4).

The pathophysiology of OHSS involves an exaggerated ovarian response to endogenous or exogenous gonadotropins, leading to the release of vasoactive mediators by granulosa cells, particularly vascular endothelial growth factor (VEGF) (5). These mechanisms lead to systemic hemodynamic alterations, characterized by arteriolar vasodilation and increased capillary permeability, which promote the translocation of fluid from the intravascular to the extravascular compartment (6). Consequently, patients may develop effective intravascular volume depletion accompanied by electrolyte disturbances, such as hyponatremia.

With the development of assisted reproductive techniques, the understanding, management, and prevention of OHSS have been extensively studied, with multiple publications and clinical guidelines available. Although the mortality risk associated with this condition is relatively low (approximately 1 in 50,000 cases), it remains a potentially serious complication (7). In contrast, spontaneous OHSS remains uncommon, even among patients with known risk factors, and is particularly rare when associated with atypical manifestations, such as concomitant hepatic involvement. Therefore, the aim of this study is to present two cases of spontaneous OHSS with hepatic involvement, describing their clinical, laboratory and imaging findings.

The study was conducted in accordance with the principles of the Declaration of Helsinki. Written informed consent was obtained from both patients. Ethical approval was granted by the Human Research Ethics Committee of Hospital Universitario Mayor–Méderi (Approval Nos. CEISH-2026031 and CEISH-2026063). The report was prepared in accordance with the CARE guidelines for case reports.

## Case presentation

### Case 1

A 21-year-old woman, G2P1, with an 8.4-week intrauterine pregnancy, was referred for moderate abdominal pain and progressive abdominal distension. On admission, she was hemodynamically stable, afebrile, and without criteria for systemic inflammatory response. Physical examination revealed a distended abdomen, deep tenderness without signs of peritoneal irritation, clinical ascites, and mild jaundice.

As part of her obstetric history, the patient reported an episode of jaundice of unclear etiology during a previous pregnancy at 11 weeks and 6 days of gestation in 2019. Imaging studies performed at that time, including magnetic resonance imaging of the abdomen and pelvis, demonstrated markedly enlarged ovaries extending to the subhepatic and subgastric regions (Figure 1). A comprehensive workup, including hepatobiliary, infectious, and autoimmune studies, yielded negative results. Magnetic resonance cholangiography was normal, and subsequent evaluation by hepatology did not reveal any additional abnormalities. During the clinical course, the patient was diagnosed with intrauterine fetal demise at 24 weeks of gestation, followed by termination of pregnancy.

**Figure 1:**
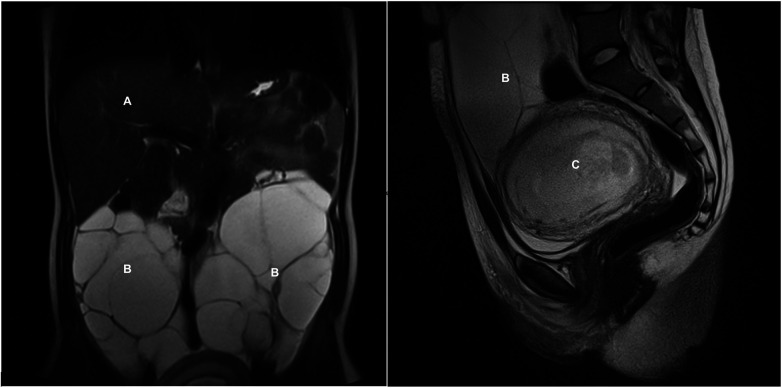
Magnetic resonance imaging of the abdomen and pelvis demonstrating marked bilateral ovarian enlargement with a multiloculated appearance, extending toward the subgastric and subhepatic regions. A gravid uterus is also visualized. The imaging findings correspond to a previous pregnancy. **(A)** Liver. **(B)** Bilaterally enlarged ovaries. **(C)** Enlarged uterus with gestational changes.

In the present pregnancy, the liver profile demonstrated persistent direct hyperbilirubinemia (3–4 mg/dL) and elevated transaminase levels, without a significant increase in gamma-glutamyl transferase (GGT) (Table 1). Initially, the biochemical pattern was predominantly cholestatic, subsequently evolving into a hepatocellular pattern. Portal Doppler ultrasound excluded portal hypertension and thrombosis, while abdominal ultrasound revealed cholelithiasis without signs of cholecystitis. Diagnostic paracentesis was performed, with ascitic fluid analysis negative for malignancy and infection. Autoimmune, infectious, and ophthalmologic studies (including evaluation to rule out Wilson disease) were unremarkable.

**Table 1:** Laboratory findings during clinical course in two cases of spontaneous ovarian hyperstimulation syndrome.

Laboratory Reports	Case 1	Case 2	Reference range
AST^a^	112–272 U/L	81–162 U/L	10–35 U/L
ALT^a^	207–578 U/L	148–346 U/L	4–36 U/L
*β* -HCG	84.558 mIU/mL	165.323 mIU/mL	13.300–254.000 mIU/mL
TSH	1.21 mIU/L	0.85 mIU/L	0.4–4.5 mIU/L
FT4	1.28 ng/dL	1.38 ng/dL	0.70–1.50 ng/dL
TB^a^	2.93–6.45 mg/dL	1.21–1.82 mg/dL	0.3–1.0 mg/dL
DB^a^	2.51–5.25 mg/dL	0.75–1.09 mg/dL	0.1–0.3 mg/dL
IB^a^	0.42–1.20 mg/dL	0.46–0.73 mg/dL	0.2–0.8 mg/dL
CA 19-9	<0.9 U/mL	12.88 U/mL	0–37 U/mL
AFP	3.3 ng/mL	2.9 ng/mL	1.31–7.89 ng/mL
CA 125	365 U/mL	31.2 U/mL	0–35 U/mL
ALP	113 U/L	100 U/L	40–147 U/L
GGT	23–26 U/L	ND	5–48 U/L

AST, aspartate aminotransferase; ALT, alanine aminotransferase; β-hCG, beta-human chorionic gonadotropin; TSH, thyroid-stimulating hormone; FT4, free thyroxine; TB, total bilirubin; DB, direct bilirubin; IB, indirect bilirubin; CA 19-9, carbohydrate antigen 19-9; AFP, alpha-fetoprotein; CA 125, cancer antigen 125; ALP, alkaline phosphatase; GGT, gamma-glutamyl transferase. ND, no data available.

a Values represent the minimum and maximum laboratory levels recorded during clinical follow-up.

The case was discussed in a multidisciplinary team meeting. The gynecology team concluded that the clinical presentation was consistent with spontaneous ovarian hyperstimulation syndrome, likely related to increased ovarian sensitivity to gonadotropins. Conservative management was initiated, with close clinical, ultrasonographic, and biochemical monitoring. The patient received comprehensive counseling and expressed her desire to continue the pregnancy. During the initial course, fetal viability was preserved, with maternal clinical stability and no progression of hepatic involvement. However, medium- and long-term follow-up could not be completed, as the patient requested voluntary discharge and did not reside in the city where care was provided, which limited continuity of outpatient monitoring and the collection of longitudinal outcome data.

### Case 2

A 23-year-old woman, G2P1, at 8.5 weeks of gestation, presented to the emergency department with a two-week history of multiple episodes of bilious vomiting associated with pressure-like abdominal pain. Prior to pregnancy, she was otherwise healthy, with regular menstrual cycles and no history of acne, hirsutism, polycystic ovary syndrome, thyroid disorders, or diabetes mellitus. Her obstetric history was notable for a term vaginal delivery five years earlier, without complications. She denied the use of medications before or during the current pregnancy.

On admission, vital signs were within normal limits. Physical examination revealed a mobile, non-tender mass in the right iliac fossa measuring approximately 9 cm. Transvaginal ultrasound demonstrated bilaterally enlarged, multiloculated ovaries, with a volume of 727 cc in the right ovary and 579 cc in the left ovary. Color Doppler evaluation showed type I vascularization, with no solid components or papillary projections identified in either ovary. Based on the overall sonographic findings, the lesions were classified as O-RADS 4. A viable embryo measuring 9.1 weeks of gestation based on crown-rump length was also observed (Figure 2). Laboratory evaluation on admission revealed elevated transaminases and hyperbilirubinemia predominantly involving the direct fraction (see Table 1). Serum *β*-hCG levels were elevated in accordance with gestational age, with negative tumor markers and normal thyroid function. Hepatobiliary ultrasound demonstrated hepatic steatosis and cholelithiasis, without signs of acute cholecystitis.

**Figure 2:**
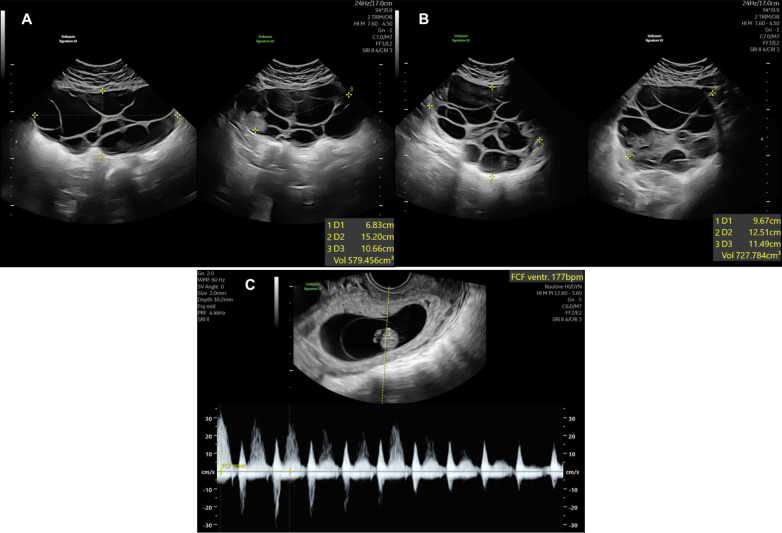
Transvaginal ultrasound demonstrating a viable intrauterine pregnancy and bilateral ovarian enlargement with a multiloculated appearance, without extension beyond the pelvic cavity. No free intraperitoneal fluid (ascites) is identified. **(A)** Left ovary. **(B)** Right ovary. **(C)** Intrauterine pregnancy with documented fetal cardiac activity.

Based on the clinical history and paraclinical findings, a diagnosis of spontaneous ovarian hyperstimulation syndrome was considered. Given the patient's clinical stability, expectant management with close laboratory and clinical follow-up was adopted. During follow-up, a first-trimester genetic screening ultrasound was performed at 12.3 weeks of gestation, showing no fetal abnormalities.

At approximately 14 weeks of gestation, the patient was readmitted to the emergency department with symptoms consistent with biliary colic. Admission laboratory tests again revealed hyperbilirubinemia and elevated transaminases, showing an upward trend compared with previously documented values. Hepatobiliary ultrasound confirmed the presence of cholelithiasis. She was subsequently evaluated by the general surgery team, which requested magnetic resonance cholangiopancreatography, revealing a 15-mm calculus impacted in the gallbladder neck.

In the context of a 14.2-week pregnancy, laparoscopic cholecystectomy was performed without complications. The patient was subsequently discharged and continued clinical follow-up under the maternal-fetal medicine team, with a favorable clinical course. However, due to logistical limitations in healthcare access, follow-up could not be maintained through delivery.

## Discussion

The presentation of spontaneous OHSS represents an extremely rare condition, with a limited number of cases described in the medical literature and no clearly established prevalence reported in available reviews.

These cases are of particular interest, as they involve patients without commonly described risk factors or comorbidities (2, 3), suggesting a hypothetical type I presentation according to the classification proposed by De Leener et al. (8). This classification categorizes spontaneous OHSS into three types based on clinical presentation and the involvement of the FSHR: type I, associated with FSHR mutations and characterized by recurrence; type II, related to elevated hCG levels, as seen in multiple pregnancies or gestational trophoblastic disease, and representing the most common form; and type III, associated with hypothyroidism (4).

Recent advances have improved the understanding of the genetic basis of OHSS, particularly in its spontaneous form. Hugon-Rodin et al. described a case of a non-pregnant woman with recurrent episodes of spontaneous OHSS, in whom, despite normal hormonal evaluation and the absence of a pituitary adenoma, a novel heterozygous mutation in the FSHR gene (c.1901 G > A) was identified, corresponding to the R634H variant located in the cytoplasmic tail of the receptor (9). Similarly, Uchida et al. reported a missense mutation (M512I) in the FSHR gene, which was not associated with hyperactivation of classical FSH-mediated signaling pathways, but rather with alterations in the PI3K/AKT pathway, suggesting an alternative mechanism in the pathophysiology of OHSS (10). These findings highlight the genetic heterogeneity of this condition and support the hypothesis that different FSHR mutations may contribute to variable clinical phenotypes, even in the absence of traditional triggering factors.

The diagnostic complexity of these cases underscores the need for a multidisciplinary approach involving general surgery, gynecologic oncology, and maternal-fetal medicine, particularly in scenarios where the absence of clear risk factors creates diagnostic uncertainty. This challenge is further compounded by the heterogeneous clinical presentation of symptoms, even in patients with the classical form of the disease (11). Ovarian malignancy is one of the most important differential diagnoses and represents a significant diagnostic challenge in this setting. In the cases presented, several findings reduced the likelihood of an underlying neoplastic process, including the possible recurrence of the condition in Case 1, the characteristics of the ascitic fluid analysis, and the absence of major sonographic features suggestive of malignancy on ultrasound examination. Baseline characteristics such as patient age, concomitant clinical manifestations including hepatic involvement, and imaging findings are fundamental elements in the diagnostic evaluation and decision-making process (12).

Hepatic involvement in patients with OHSS has been described, primarily in the context of assisted reproductive techniques, with one of the earliest cases reported in 1988 (13). This clinical manifestation should be carefully considered within the differential diagnosis. In terms of severity, mild OHSS may present with abdominal distension, nausea, vomiting, or diarrhea, whereas moderate, severe, and critical forms can be associated with symptomatic ascites, hydrothorax, oliguria or anuria, renal failure, hepatic dysfunction with elevated transaminases, thromboembolic events, acute respiratory distress syndrome, and sepsis. In this context, hepatic involvement should not be regarded solely as an atypical manifestation, but rather as part of the clinical spectrum of the disease, and therefore should be systematically considered in the diagnostic approach and comprehensive evaluation of these patients (14).

The pathophysiology of hepatic dysfunction in OHSS is not fully understood. Proposed mechanisms include increased vascular permeability resulting in hepatic edema, as well as microvascular thrombosis that may compromise hepatic perfusion and lead to tissue ischemia (15). The coexistence of other hepatobiliary disorders may represent an important diagnostic challenge and potential confounding factor during the evaluation of liver involvement in these patients. Nevertheless, several clinical, laboratory, and imaging features can help distinguish OHSS-related hepatic dysfunction from alternative hepatic and biliary conditions. The main differential diagnostic considerations and their distinguishing characteristics are summarized in Table 2.

**Table 2:** Clinical, laboratory, and imaging features of common causes of hepatic dysfunction in pregnancy and OHSS.

Feature	OHSS	Hepatobiliary disorders	Intrahepatic cholestasis of pregnancy	HELLP syndrome
Clinical features				
Jaundice	**++**	**+++**	**+**	**+**
Abdominal pain	**++**	**+++**	**±**	**+++**
Fever	**−**	**++**	**−**	**−**
Hypertension	**−**	**−**	**−**	**+++**
Laboratory findings				
AST	**++**	**+++**	**++**	**+++**
ALT	**++**	**+++**	**++**	**+++**
TB	**++**	**+++**	**+**	**+**
DB	**+**	**+++**	**+**	**+**
IB	**+**	**+**	**+**	**++**
GGT	**+**	**+++**	**±**	**+**
ALP	**±**	**+++**	**+**	**±**
Imaging findings				
Biliary duct dilatation	**−**	**+++**	**±**	**−**
Findings suggestive of pancreatitis	**−**	**+++**	**−**	**−**
Hepatic rupture	**−**	**−**	**−**	**+**

OHSS, ovarian hyperstimulation syndrome; AST, aspartate aminotransferase; ALT, alanine aminotransferase; TB, total bilirubin; DB, direct bilirubin; IB, indirect bilirubin; ALP, alkaline phosphatase; GGT, gamma-glutamyl transferase. Frequency scale: +++, common/frequent finding; ++, moderately frequent finding; +, uncommon finding; ±, may be present; −, usually absent.

In the setting of spontaneous OHSS, no direct associations have been described between genetic risk patterns and hepatic or biliary tract disorders. In the present cases, abdominal pain was the predominant clinical manifestation, and the initial diagnostic approach was guided by abnormalities in liver function tests. The presence of cholelithiasis in both patients is noteworthy; however, the initial clinical and imaging findings were not consistent with acute cholecystitis, although the second patient ultimately required surgical management. These observations underscore the importance of considering spontaneous OHSS in the differential diagnosis when abdominal symptoms coexist with hepatic abnormalities and suggestive ultrasound findings.

From the patient perspective, both individuals expressed satisfaction with gaining a better understanding of their condition during medical care and conveyed interest in further research to deepen knowledge of this entity.

### Study limitations

Among the main limitations of the present report is the inability to perform complete follow-up of either patient through delivery. Long-term maternal and fetal outcomes were not available due to loss to follow-up, precluding assessment of perinatal morbidity, postpartum outcomes, and recurrence risk.

Additionally, no complementary genetic studies were available at our institution to confirm the presence of FSHR gene variants (e.g., R634H and M512I), representing a missed opportunity for a more precise etiological characterization and potential classification as type I OHSS. Nevertheless, these findings open avenues for future research focused on the clinical and epidemiological characterization of spontaneous OHSS in the absence of additional risk factors, as well as the investigation of potential genetic predispositions. Furthermore, they highlight the need for additional studies exploring hepatic involvement in OHSS and its possible association with concomitant hepatobiliary disorders.

## Conclusion

Spontaneous OHSS is a rare condition that should be considered in pregnant patients presenting with abdominal symptoms, ovarian enlargement, and abnormal liver function tests, even in the absence of assisted reproductive techniques. Hepatic involvement should be recognized as part of its clinical spectrum, and its coexistence with biliary findings may complicate the diagnostic approach. These cases highlight the importance of a comprehensive and multidisciplinary evaluation in atypical presentations and underscore the need for further studies to better characterize the epidemiological behavior of the disease and its associated genetic mechanisms.

## Data Availability

The original contributions presented in the study are included in the article/Supplementary Material, further inquiries can be directed to the corresponding author/s.
